# The Prevalence of Restless Legs Syndrome Among Pregnant Women in Saudi Arabia

**DOI:** 10.7759/cureus.42883

**Published:** 2023-08-03

**Authors:** Saud Alnaaim, Fatimah Alghirash, Abdulelah Alenzi, Mohammad Owaidh Abu Zahirah, Thekra Tashari, Faisal Hakami, Rehaf Alsharif

**Affiliations:** 1 Neurology, King Faisal University, Hofuf, SAU; 2 Medicine, King Faisal University, Hofuf, SAU; 3 Neurology, Rumah General Hospital, Riyadh, SAU; 4 Neurology, King Faisal Hospital, Makkah, SAU; 5 Medicine, Jazan University, Jazan, SAU; 6 Medicine, Umm Al-Qura University, Makkah, SAU

**Keywords:** saudi arabia, risk factors, restless legs syndrome, pregnancy, prevalence

## Abstract

Introduction: Pregnancy has been associated with restless legs syndrome (RLS). RLS is underdiagnosed in most countries. The purpose of this study was to assess the prevalence of RLS among Saudi pregnant women.

Methods: This cross-sectional study included 459 pregnant women from all Saudi provinces between December 2022 and March 2023. A structured online questionnaire assessing demographic and pregnancy characteristics, medical conditions, sleep quality, and RLS symptoms was conducted.

Results: The prevalence of RLS was 26.6% among the participants. Calcium deficiency was significantly associated with RLS (adjusted odds ratio (OR)=2.16, 95% confidence interval (CI)=1.2-3.9, p=0.01), but there was no significant association between RLS and vitamin D and iron deficiencies (p>0.05), according to multivariate binary logistic regression analysis. Moreover, RLS increased the risk of insomnia and frequent waking up (adjusted OR=4.95, 95% CI=2.50-9.83, p<0.001, and adjusted OR=2.87, 95% CI=1.55-5.32, p=0.001, respectively).

Conclusion: RLS is common among pregnant women in Saudi Arabia. The study indicates that RLS during pregnancy is linked to calcium deficiency and negatively affects sleep quality.

## Introduction

Restless legs syndrome (RLS), commonly referred to as Willis-Ekbom disease, is a sensorimotor neurological disorder that mostly affects the legs [1]. The diagnosis of RLS depends on five well-defined criteria: (i) the urge to move the legs whether it was with or without the abnormal sensation, (ii) worsening of symptoms with rest, (iii) improvement with activity, such as walking or stretching, (iv) worsening of symptoms at night due the effect of the circadian pattern, (v) and being independent of other medical or behavioral conditions [2].

The prevalence of RLS in pregnant women is at least two to three times higher than in the general population, 11%-34% and 2%-10%, respectively [3-5]. The lowest rate of RLS was reported in Western pregnant women [6]. In contrast, the prevalence of RLS during pregnancy has been reported to be up to 30% in Eastern Mediterranean countries [5]. Despite these records, RLS is still underdiagnosed and undertreated due to unawareness of RLS in pregnant women and their consideration of it as a normal healthy condition that appears with pregnancy [7].

The possible pathophysiological mechanism of occurrence of RLS in pregnancy is metabolic alterations, such as iron and folate deficiency, as well as hormonal alterations due to the increase of prolactin, estrogen, and progesterone during late pregnancy [3]. Vitamin D deficiency and calcium metabolism may also play a role [8]. Some factors, such as smoking, alcohol, and caffeine consumption, have been suggested to trigger RLS during pregnancy [5].

The highest degree of severity of RLS symptoms occurs in the third trimester, with a tendency to alleviate during delivery [3]. RLS was found to be associated with some late pregnancy complications, including preeclampsia, threatened miscarriage, intrauterine growth retardation, and increased incidence of cesarean section [5,8]. Furthermore, RLS has been associated with decreased quality of life, poor sleep quality, poor daytime function, and excessive daytime sleepiness in pregnant women [1,9].

Given the negative relationship of RLS to health outcomes in pregnancy, estimating its prevalence and identifying its related conditions is essential. Therefore, the present study aims to measure the prevalence of RLS among pregnant women in Saudi Arabia.

## Materials and methods

An observational cross-sectional study was conducted to measure the prevalence of restless legs syndrome in pregnant women from all provinces of Saudi Arabia between 2022 and 2023. The selection of the study’s population was done using a convenience sampling method. Exclusion criteria comprised women who are not Saudi and not pregnant and those who refused to participate. A total of 385 participants were determined as a sample size according to a confidence level of 95% and a margin of error of 5%.

Data were collected through an online questionnaire that included demographic information, other parameters such as smoking and presence of chronic or psychiatric diseases, sleep quality, and pregnancy characteristics, including the duration of pregnancy, number of pregnancies, gastrointestinal-related diseases, and common nutrient deficiencies during pregnancy. RLS was diagnosed based on an Arabic version of the International Restless Legs Syndrome Study Group (IRLSSG) questionnaire [2]. The IRLSSG questionnaire consists of five criteria: (i) a desire to move the legs, (ii) exacerbation during rest, (iii) improvement by activity, (iv) predominance of symptoms at night, (v) and being unrelated to other medical or behavioral disorders. Individuals who confirmed the presence of all five criteria were diagnosed with RLS.

Statistical analysis was conducted using Statistical Package for the Social Sciences (SPSS) version 25 (IBM SPSS Statistics, Armonk, NY, USA). Descriptive statistics, including frequencies and percentages, were generated to summarize categorical variables of interest. To compare demographic characteristics and medical variables with the presence of RLS, Pearson’s chi-square and Fisher’s exact tests were employed. Additionally, binary logistic regression analysis was conducted to perform further investigation. A p-value of <0.05 was utilized as the statistical significance level.

## Results

Sociodemographic and pregnancy characteristics

Table 1 summarizes the study subjects’ sociodemographic and pregnancy characteristics, including age, educational level, smoking status, presence of chronic and psychiatric disorders, pregnancy phase, sleep-related issues, pregnancy frequency, and diagnosed conditions. The study included 459 subjects, with the majority falling in the age range of 26-35 years (48.1%). Most of the subjects had a university degree or higher (76.3%) and were non-smokers (93.9%). A significant proportion of the subjects reported having chronic disorders (12.9%) and psychiatric disorders (6.8%). Regarding pregnancy-related factors, the majority of the subjects were in the second or third trimester of pregnancy (37.5% and 39.4%, respectively), and a significant proportion reported experiencing insomnia (44.7%) and frequent waking up during the night (37.3%). Most of the subjects had been pregnant two to five times (55.7%). Finally, Table 1 includes information on diagnosed conditions, with iron deficiency being the most commonly reported condition (33.3%), followed by vitamin D deficiency (25.5%), gestational diabetes mellitus (GDM) (10.2%), and preeclampsia/eclampsia (3.3%).

**Table 1:** Sociodemographic and pregnancy characteristics

Sociodemographic characteristic	Frequency (%) (N=459)
Age (years)	
18-25	112 (24.4%)
26-35	221 (48.1%)
36-45	116 (25.3%)
More than 45	10 (2.2%)
Educational level	
Not educated	4 (0.9%)
Primary school	6 (1.3%)
Intermediate school	28 (6.1%)
Secondary school	71 (15.3%)
University or higher	350 (76.3%)
Smoker	
No	431 (93.9%)
Yes	28 (6.1%)
Chronic disorder	
No	400 (87.1%)
Yes	59 (12.9%)
Psychiatric disorder	
No	428 (93.2%)
Yes	31 (6.8%)
Pregnancy phase	
First trimester	106 (23.1%)
Second trimester	172 (37.5%)
Third trimester	181 (39.4%)
Having insomnia	
No	254 (55.3%)
Yes	205 (44.7%)
Frequent waking up during the night	
No	288 (62.7%)
Yes	171( 37.3%)
Pregnancy frequency	
First time	164 (35.8%)
2-5 times	255 (55.7%)
More than five times	39 (8.5%)
Diagnosis	
Preeclampsia/eclampsia	15 (3.3%)
Gestational diabetes	47 (10.2%)
Vitamin D deficiency	117 (25.5%)
Calcium deficiency	86 (18.7%)
Iron deficiency	153 (33.3%)

IRLSSG criteria among the study subjects

The frequency and percentage of RLS diagnostic criteria fulfillment among the study subjects, as per the IRLSSG criteria, are displayed in Table 2. Among the 459 study subjects, 129 (28.1%) did not meet any of the five IRLSSG diagnostic criteria for RLS, while 32 (7%) met only one criterion, and 27 (5.9%) met two criteria. The number of subjects meeting three, four, or all five criteria was 64 (13.9%), 85 (18.5%), and 122 (26.6%), respectively. It is worth mentioning that participants who met all five criteria were diagnosed with RLS, which means that 26.6% of the study subjects were diagnosed with this condition.

**Table 2:** IRLSSG criteria among the study subjects *Participants who had met all five criteria are diagnosed with restless legs syndrome. IRLSSG: International Restless Legs Syndrome Study Group

Number of fulfilled IRLSSG diagnostic criteria	Frequency	Percentage
No criteria have been met	129	28.1%
Only one criterion	32	7%
Two criteria	27	5.9%
Three criteria	64	13.9%
Four criteria	85	18.5%
Five criteria*	122	26.6%

The relationship of RLS with different study subjects’ characteristics

Table 3 presents the results of Pearson’s chi-square and Fisher’s exact tests for the assessment of RLS among different sociodemographic and medical variables of the study subjects. The analysis found that age, educational level, smoking status, the presence of chronic and psychiatric disorders, and preeclampsia were not significantly associated with RLS (p>0.05). However, suffering from insomnia, frequent waking up during the night, pregnancy frequency, vitamin D deficiency, calcium deficiency, and iron deficiency were significantly associated with RLS (p<0.05).

**Table 3:** Relationship of RLS with different study subjects’ characteristics *Alpha criterion was set as 0.05 or fewer, which is considered a significant value. RLS: restless legs syndrome, NS: not significant

Sociodemographic characteristic	Pregnant with RLS		p-value*
	Yes (frequency (%))	No (frequency (%))	
Age (years)			
18-25	22 (18%)	90 (26.7%)	NS
26-35	58 (47.5%)	163 (48.4%)	
36-45	37 (30.3%)	79 (23.4%)	
More than 45	5 (4.2%)	5 (1.5%)	
Educational level			
Not educated	0 (0%)	4 (1.2%)	NS
Primary school	1 (0.8%)	5 (1.5%)	
Intermediate school	5 (4.1%)	23 (6.8%)	
Secondary school	21 (17.2%)	50 (14.8%)	
University or higher	95 (77.9%)	255 (75.7%)	
Smoker			
No	114 (93.4%)	317 (94.1%)	NS
Yes	8 (6.6%)	20 (5.9%)	
Chronic disorder			
No	113 (92.6%)	315 (93.5%)	NS
Yes	9 (7.4%)	22 (6.5%)	
Psychiatric disorder			
No	106 (86.9%)	294 (87.2%)	NS
Yes	16 (13.1%)	43 (12.8%)	
Suffering from insomnia			
No	20 (16.4%)	234 (69.4%)	<0.001*
Yes	102 (83.6%)	103 (30.6%)	
Frequent waking up during the night			
No	31 (25.4%)	257 (76.3%)	<0.001*
Yes	91 (74.6%)	80 (23.7%)	
Pregnancy frequency			
First time	36 (29.5%)	128 (38.1%)	0.04*
2-5 times	70 (57.4%)	185 (55.1%)	
More than five times	16 (13.1%)	23 (6.8%)	
Pregnancy phase			
First trimester	22 (18%)	84 (24.9%)	NS
Second trimester	44 (36.1%)	128 (38%)	
Third trimester	56 (45.9%)	125 (37.1%)	
Preeclampsia			
No	117 (95.9%)	327 (97%)	NS
Yes	5 (4.1%)	10 (3%)	
Gestational diabetes			
No	108 (88.5%)	304 (90.2%)	NS
Yes	14 (11.5%)	33 (9.8%)	
Vitamin D deficiency			
No	78 (63.9%)	264 (78.3%)	0.002*
Yes	44 (36.1%)	73 (21.7%)	
Calcium deficiency			
No	81 (66.4%)	292 (86.6%)	<0.001*
Yes	41 (33.6%)	45 (13.4%)	
Iron deficiency			
No	65 (53.3%)	241 (71.5%)	<0.001*
Yes	57 (46.7%)	96 (28.5%)	

Specifically, 83.6% of subjects who suffered from insomnia had RLS, compared to only 16.4% of those who reported not having insomnia (p<0.001). Similarly, 74.6% of subjects who frequently woke up during the night had RLS, compared to only 25.4% of those who did not report this problem (p<0.001). The results suggest that a lower pregnancy frequency is associated with a higher prevalence of RLS. Specifically, the prevalence of RLS was highest among subjects who had been pregnant two to five times (57.4%), followed by those who had been pregnant for the first time (29.5%), and then those who had been pregnant more than five times (13.1%) (p=0.04). Regarding medical conditions, the prevalence of RLS was significantly higher among subjects with vitamin D deficiency (36.1%), calcium deficiency (33.6%), and iron deficiency (46.7%) compared to those without these deficiencies (p=0.002, p<0.001, and p<0.001, respectively).

Regarding pregnancy frequency and medical conditions, the logistic regression did not find a significant association between RLS and pregnancy frequency or vitamin D and iron deficiencies (p>0.05). However, the presence of calcium deficiency was significantly associated with RLS (adjusted odds ratio (OR)=2.16, 95% confidence interval (CI)=1.2-3.9, p=0.01) as presented in Table 4.

**Table 4:** Association between RLS and statistically significant variables in Table 3 *Alpha criterion was set as 0.05 or fewer, which is considered a significant value. RLS: restless legs syndrome, NS: not significant, OR: odds ratio, CI: confidence interval

Predicted variable	Adjusted OR	95% CI	p-value
Pregnancy frequency			
First time	Reference		
2-5 times	1.16	0.67-2	NS
More than five times	1.87	0.77-4.52	NS
Vitamin D deficiency			
No	Reference		
Yes	0.98	0.56-1.77	NS
Calcium deficiency			
No	Reference		
Yes	2.16	1.2-3.9	0.01*
Iron deficiency			
No	Reference		
Yes	1.27	0.76-2.11	NS

The association between RLS and sleep disturbances

In Table 5, the results of multivariate binary logistic regression analysis conducted to predict sleep disorders among pregnant women with or without RLS are presented. The analysis indicated a significant association between RLS and insomnia, with pregnant women with RLS being approximately five times more likely to experience insomnia (adjusted OR=4.95, 95% CI=2.50-9.83, p<0.001) compared to their counterparts without RLS. Additionally, the regression analysis revealed that those with RLS had approximately three times higher likelihood of frequently waking up during the night (adjusted OR=2.87, 95% CI=1.55-5.32, p=0.001) due to the influence of RLS.

**Table 5:** Association between RLS and sleep disturbances Adjusted to statistically significant variables in Table 3 *Statistically significant value RLS: restless legs syndrome, OR: odds ratio, CI: confidence interval

RLS (predicted variable)	Suffering from insomnia			Frequent waking up during the night		
	Adjusted OR	95% CI	p-value	Adjusted OR	95% CI	p-value
Without	Reference			Reference		
With	4.95	2.50-9.83	<0.001*	2.87	1.55-5.32	0.001*

## Discussion

RLS is a common but frequently under-recognized and undertreated neurological disorder characterized by an irresistible urge to move the legs during rest [10]. RLS is estimated to affect up to 10% of the general population, although pregnant women are thought to be at a higher risk [11]. A review of the literature revealed that the prevalence of RLS among pregnant women ranges from 10% to 34% [7,8,11], including 22%, 14%, 30%, and 20% of pregnant women in the European, Western Pacific, Eastern Mediterranean, and Americas regions, respectively [5]. The methodology, study populations, and gestational age at the time of the assessment have all been suggested as potential explanations for the discrepancies in the reported prevalence of RLS in pregnancy [5]. The current study shows that 26.6% of 459 Saudi pregnant women met the diagnostic criteria for RLS, which is consistent with the results of other previous studies.

RLS is common in pregnancy and has further been linked to advanced gestational age. Al Shidhani et al. found a significantly increased rate of RLS in the third trimester (41%) compared to the first trimester (15.7%) [6], similar to the current results, although they are not statistically significant. The finding regarding the role of frequent pregnancy on RLS did not show a relationship in the present study, which is consistent with an Omani study [6].

While a study by Liu et al. has found an increased prevalence of RLS with age during pregnancy [12], numerous studies have shown no correlation between age and RLS [13-15]. Their finding is similar to the present result. In addition, this study did not show any significant association between smoking and RLS. This finding was also reported in the literature [14,15]. However, a study by Güler et al. has found that smoking increases the likelihood of developing RLS, with a prevalence of 9.8% [10].

According to studies, RLS in relation to gestational-related diseases, such as GDM, pregnancy-induced hypertension (PIH), and preeclampsia, have yielded varying results. Innes et al. found that the risk of RLS is five times higher in pregnant women with GDM [16]. Researchers also found an association between RLS and PIH, with over 24% of the participants having a positive history of PIH [17]. A significant proportion of RLS patients reported having preeclampsia in a study by Ramirez et al. (p=0.03) [18]. However, in a cohort study that assessed 1,563 pregnant women, hypertension and diabetes had similar frequencies in the RLS and control groups [1]. The present study also shows no significant association between GDM and preeclampsia and RLS. Moreover, there was no significant difference between chronic and psychiatric diseases and RLS in this study.

During pregnancy, it is not uncommon to have nutrient deficiencies. Sağlam et al. found a relationship between vitamin D deficiency and RLS, in which 58.2% of pregnant women with vitamin D deficiency reported symptoms of RLS [19]. Alteration of levels of dopaminergic neurons, which are typically protected by vitamin D, have been utilized to clarify how vitamin D deficiency contributes to the development of RLS. Furthermore, patients with RLS have elevated levels of vitamin D binding protein in their cerebrospinal fluid, which further supports the involvement of vitamin D [19]. In the present study, although vitamin D deficiency was higher in pregnant women with RLS than in those without RLS, no statistically significant association was found between the two groups. The three- to fourfold increase in iron needs during pregnancy has been linked to the occurrence of RLS in this population [20]. Moreover, the iron supplementation that is given prior to pregnancy has been reported to alleviate the symptoms of RLS [8]. Despite these studies, the current result revealed that iron deficiency was not a significant indicator of RLS (p>0.05). In a study that identified the risk factors of RLS among patients undergoing hemodialysis, serum calcium concentrations were associated with the severity of RLS [21]. This result is in line with the current study’s finding, which showed that calcium deficiency was a strong predictor of RLS (p=0.01).

RLS has a negative impact on the sleep quality of pregnant women. A population-based cohort study found that the sole factor of the long-increasing trajectory and decreased sleep duration is RLS [22]. A recent comparative descriptive study found that 92.3% of pregnant women with RLS experienced fatigue and 67.3% desired to sleep during the day [23]. Kızılırmak et al. determined RLS as the third most common cause of insomnia in pregnancy [24]. Among pregnant women with RLS in this study, 83.6% report insomnia, and 74.6% report frequent waking up during the night, indicating approximately a fivefold increased risk of insomnia and a threefold increased risk of recurrent waking up in this population.

The current study is based on the IRLSSG diagnostic criteria, addressing the risk and prevalence of RLS among pregnant Saudi women. Additionally, it assessed the link between RLS and sleep disorders, as well as certain medical conditions, highlighting the need for better early detection and management of RLS. The major strength of this study is that it measures the prevalence of RLS in all provinces of Saudi Arabia, with over 456 participants. This study has limitations, including the absence of clinical assessments. The survey is self-reporting, and both recall and response bias are possible. The use of procedures and blood tests to support the diagnosis of RLS is lacking.

## Conclusions

RLS during pregnancy is high in Saudi women, with approximately a quarter of the participants meeting the five diagnostic criteria of RLS. The study revealed that RLS during pregnancy was strongly linked to calcium deficiency. However, no significant association was found with other biochemical parameters, such as vitamin D and iron, gestational-related diseases, smoking, and age. A significant relationship was found between RLS and poor sleep quality in pregnant women. Based on the results of this study, more health campaigns and volunteer efforts to educate pregnant women about this condition are necessary for early detection and treatment. Physicians should be also aware of the importance of the screening of RLS with an evaluation of sleep and quality of life during the antenatal visit. Further studies are needed to evaluate the pathophysiology and etiology of RLS during pregnancy in terms of evidence-based medicine.
